# Unlocking the Recovery Potential: JMJD3 Inhibition-Mediated SAPK/JNK Signaling Inactivation Supports Endogenous Oligodendrocyte-Lineage Commitment Post Mammalian Spinal Cord Injury

**DOI:** 10.1007/s11064-020-03210-z

**Published:** 2021-01-11

**Authors:** Zhang Bo-Yin, Zhu Qingsan, Ma Yihang, Yang Fan, Zhu Yuhang, Chang Pengyu

**Affiliations:** 1 Orthopedics Surgery Department, China-Japan Union Hospital of Jilin University, Changchun, 130033 Jilin China; 2 Radiotherapy Department, The First Bethune Hospital of Jilin University, Changchun, 130021 Jilin China

**Keywords:** Spinal cord injury, Epigenetic modification, JMJD3, H3K27me3, Demethylation

## Abstract

Spinal cord injury (SCI) induced catastrophic neurological disability is often incurable at present. The injury triggered immediately oligodendrocytes loss and overwhelming demyelination are regarded as an insurmountable barrier to SCI recovery. To date, effective strategy to promote the endogenous oligodendrocytes replacement post SCI remains elusive. Epigenetic modifications are emerging as critical molecular switches of gene expression in CNS. However, the epigenetic mechanisms underlying oligodendrogenesis post SCI yet to be discovered. In this study, we report that H3K27me3 demethylase JMJD3 exists as a pivotal epigenetic regulator which manipulates the endogenous oligodendrogenesis post SCI. We found that JMJD3 inhibition promotes the oligodendrocyte linage commitment of neural stem/progenitor cells (NPCs) in vitro and in vivo. Moreover, we demonstrated that JMJD3 inhibition mediated SAPK/JNK signaling inactivation is functionally necessary for endogenous oligodendrocyte-lineage commitment post SCI. Our results also suggested that JMJD3 is downstream of SAPK/JNK pathway, and capable of translates SCI induced SAPK/JNK signaling into epigenetic codes readable by spinal cord endogenous NPCs. Taken together, our findings provide novel evidence of JMJD3 mediated oligodendrocyte-lineage commitment orchestration post SCI, which would be a potential epigenetic approach to induce the mature mammalian endogenous recovery.

**Supplementary information:**

The online version of this article (10.1007/s11064-020-03210-z) contains supplementary material, which is available to authorized users.

## Introduction

Spinal cord injury (SCI) is a catastrophic neurotraumatic event which results in permanent neurological disability. With the development of transportation and construction in recent years, the incidence rate of SCI elevated progressively. It is estimated that the worldwide annual arising of new SCI cases is more than 760,000 approximately [1]. Unfortunately, SCI triggered devastating neurological symptoms is still incurable [2].

Oligodendrocytes (OLs) orchestrate the axon myelin sheaths in central nervous system (CNS), which guarantee the neural signal rapid transmission and axon metabolic support [3]. Nevertheless, OLs are extremely vulnerable to the SCI strike [4]. Primary neurotrauma usually triggers serious OLs loss and diffused demyelination immediately, which impairs the axon conduction capacity and survival eventually [5, 6].

Emerging evidence demonstrates that the spontaneous endogenous cell genesis post mammalian SCI provide a silver lining for SCI translational studies [7–9]. Nevertheless, the potential of reprogramming and oligodendrogenesis of adult spinal cord neural stem/progenitor cells (NPCs) is limited [10, 11]. In SCI lesion, oligodendrocyte-lineage commitment of NPCs is the prerequisite for the endogenous OLs replacement, which is definitely of pivotal significance for neurological recovery [12]. To date, effective strategy to augment the potential remains to be developed.

Epigenetic modification independent of DNA sequence transformation has been proved to manipulate cell fate determination and lineage commitment precisely [13, 14]. Tri-methylation of lysine27 on histone3 (H3K27me3) serves as a typical repressive epigenetic hallmark which coordinates the gene expression by maneuvering chromatin accessibility around promoter sites reversibly [15]. The Jmjc-domain containing histone demethylase(JMJD3) is a demethylase of H3K27me3, which acts as a pivotal regulator to revise the gene expression status at the chromatin level [16]. However, the roles JMJD3 played in oligodendrogenesis post SCI remains unknown.

The present study, we reported that H3K27me3 demethylase JMJD3 regulates the oligodendrogenesis post SCI. Inhibition of JMJD3 promotes the oligodendrocyte-lineage commitment of NPCs. Furthermore, JMJD3 is downstream of SAPK/JNK pathway, and capable of translates SCI induced SAPK/JNK signaling into epigenetic codes readable by spinal cord NPCs.

## Material and Method

### Animals

All animals used in the experiments were handled according to the guideline of the Institutional Animal Care and Use Committee of Jilin University. The 8 to 12-week-old adult female C57/BL6 mice (weighing from 30 to 40 g) were purchased from Animal Facility of Vital River Laboratory (Beijing, China).

### Reagents and Antibodies

The JMJD3 antibodies were from CST (#3457) and Abcam(ab38113). The #3457 was for immunoblot assay and ab38113 was for IHC staining. The Olig2 antibody was from Abcam(ab109186). The GFAP antibody was from Abcam(ab7260). The PDGFR-a antibody was from Abcam(ab203491). FACS antibodies was from BD, CD133 (566596), CD140a (740148). The H3K27me3 antibody was from CST (#9733). The Histone3 antibody was from CST (#4499). The antibodies for SAPK/JNK signaling study were from CST, P-c-fos(#5348), P-JNK(#4668), JNK(#9252), P-c-jun(#91952), c-jun(9165). The actin antibody was from Abcam(ab6276). The specific inhibitors were from MedChemeExpress, GSK-J4(HY-15648b), SP600125(HY-10241), Vinblastine (HY-13780), Retinoic acid (HY-14649).

bFGF and EGF were from Peprotech (45033, 31509). DMEM/F12, N2, B27and TrypLE express was from Gibco. The collagenase A was from ROCHE (10103578001). Protease and Phosphatase inhibitor cocktail kit was from Sigma Aldrich (P8340, P2850). IHC DAB kit was from Abcam(ab94665). ECL kits were from Abcam(ab133406).

### In Vitro Cell Culture

#### Primary Culture of Spinal Cord NPCs and Identification

The P0 mice spinal cord NPCs were used for lineage commitment and signaling pathway screening assays. Briefly, the P0 mice spinal cord was carefully dissected out and cut into fragments under surgical microscope. The fragments were digested at 37 °C with collagenase A (1 mg/ml, 90 min), followed by trypsin–EDTA (0.05%, 15 min). The digested fragments were washed with complete medium (DMEM/F12 medium supplemented with 1% Glutamax, 100x B27, 100xN2, 20 ng/ml EGF, 20 ng/ml bFGF) for three times and then mechanically dissociated with 1 ml pipette tip. After the centrifuge, cells were re-suspended and plated onto 6 well plates at a density of 1 × 104/ml for primary neurospheres suspension culture. For NPCs identification, the surface marker Nestin expression of neurospheres was detected by immunofluorescent staining. For lineage commitment assay, NPCs neurospheres were digested into single cells and then seeded onto Martigel-coated coverslips and cultured with differentiation medium (without EGF, bFGF, but supplemented with 2%FBS). For screen experiment of SAPK/JNK signaling pathway, NPCs neurospheres were digested into single cells and then seeded onto Martigel-coated coverslips and cultured with conditioned medium (differentiation medium supplemented with Vinblastine at 20 uM, SP600125 at 20 uM, GSK-J4 at 10 uM, Retinoic acid at 2 uM).

#### C17.2 NPCs Cell Line Culture

For proliferation, murine multipotent neural stem/progenitor cell line C17.2 were seeded at a density of 1 × 105/ml and incubated for 48 h in complete medium (DMEM supplemented with 10%FBS) until reaching 70–80% confluence. Thereafter, the complete medium was replaced with starved medium for differentiation (FBS free DMEM, 20 ng/ml NGF). For lineage commitment assay, GKS-J4 was added in starved medium at optimized working concentration (10 uM) of JMJD3 inhibition group. After 2 days culture, C17.2 cells were harvested for further protein detection.

#### Spinal Cord Injury Model

The mice thoracic hemi-transection SCI model (T9 level, right side) was established according to previous studies [17]. Briefly, the mice were deeply anesthetized with 0.1 ml/10 g of 5% chloral hydrate via intraperitoneal injection. After the OR site the shaving and skin disinfected, a posterior back midline incision was surgical exposed. The T8–10 spinal cord segments (the apex point of prone position) were exposed after paraspinal muscles dissection and laminectomy with bone scissors. Once the cord dorsal middle vessel can be clearly recognized, the right-side hemi-transection of the spinal cord was performed fully penetrated from dorsal to ventral with fine microsurgical scissors. Injury site antibiotic injection and manual bladder expression were administrated post the operation. All surgical procedures were performed under the surgical microscope.

#### Reagents Injection

The reagents injection model was established according to the diagrams of Fig. S4–5. Briefly, the reagents were injected in either intraperitoneally or subcutaneously around the T9 spine segment with a fine and precision syringe (1 ml). For the intraperitoneal injection, reagents were injected into the lower left quadrant of abdomen gently. For the subcutaneous injection, 3 points were selected around the T9 posterior midline, 0.5 cm paraspinal points (left and right) and T9 spinous process point. For naive JMJD3 inhibition animal model, the mice received GSK-J4 injection 5 day intraperitoneally and 7 days subcutaneously. For JMJD3 inhibition SCI model, the mice received a T9 right side hemi-transection post 5 days intraperitoneal GSK-J4 injection. Afterwards, the SCI model received GSK-J4 subcutaneous injection for 7 consecutive days. DMSO injection mice were served as GSK-J4-free controls. All Reagents injection was performed once a day at the concentration of 100 mg/Kg (Dissolved in 10% DSMO).

#### Tissue Preparation and Immunohistochemistry

Mice in each group were anesthetized transcardially perfused with 4% ice-cold PFA. The spinal cords were harvested (0.5 cm around epicenter) and post-fixed in 4% PFA for 6 h. Dehydration of PFA fixed sample is achieved by immersion in increasing concentrations of alcohol. Later, 4-um-thick paraffin-embedded sections were prepared for IHC staining, and then these sections were dewaxed and rehydrated for following staining. After antigen retrieval and non-specific antigens blocking, the sections were incubated with primary and secondary antibodies in sequence. Finally, the IHC DAB kit was used to identify positive cells in spinal cord tissue.

### Western Blot Analysis

Spinal cord tissues (0.5 cm around epicenter) or cultured NPCs were collected and lysed using the RIPA buffer. The sample was prepared with protease and phosphatase inhibitor cocktail reagents. Heat-denatured protein samples were separated by SDS-PAGE gel electrophoresis and then transferred onto PVDF membranes. After blocking with 10% non-fat milk, membranes were incubated with primary antibodies(4 °C, overnight), followed by HRP-linked secondary antibodies(room temperature, 1 h). Finally, chemiluminescent ECL reagent was used for the bands detection.

### Immunofluorescent Assay

Cultured NPCs were fixed with 4%PFA for 20 min at room temperature. Fixed cells were washed with PBS and blocked in blocking buffer (2% BSA, 0.1% Triton X-100, 0.1% sodium azide in PBS). Primary and secondary antibodies were diluted with the blocking buffer and incubated for 1 h each at room temperature. After the incubation, the coverslips were rinsed and mounted for observation. The fluorescence images of NPCs were captured with an inverted florescence microscope, and the 488, 568 wavelength channels were used for according to florescence labels of secondary antibodies.

### FACS

Briefly, the spinal cords were harvested 7DPI (0.5 cm around epicenter). The periventricular zone tissues of spinal cord segment were gently separated under surgical microscopy, and the dural mater and white matter were removed. Carefully cut tissue into fragments under microscope and then dissociated into single cell suspension. Cell pellets were incubated with fluorescent-conjugated antibodies (CD133 and CD140a) and washed with PBS. Thereafter, the prepared cells were re-suspended and analyzed with the FACS flow cytometer (BD, Aria II). Fluorescent intensities for cells were point-plotted on two-axis graphs using FlowJO.

### Quantification and Statistical Analysis

For the fluorescence intensity quantification, the immunofluorescent stained NPCs were captured as above (Immunofluorescent assay). The fluorescence intensity of unmerged 488-channel (Olig2+) picture was measured by Image-J software (v.1.8.0). Briefly, the images were transferred into 8 bit gray level and inverted into negative image format. Then the range of threshold was set upped base on each NPCs colony. Finally, the fluorescence intensity value of selected area was measured. For the Olig2 positive cells and tube-like structures quantification, IHC stained slides were prepared as above (Tissue preparation and immunohistochemistry). Then Olig2 positive cells and tube-like structures (tubes without stained nuclear) on the slides were counted and calculated. The data are expressed as mean ± standard deviation (SD) for the indicated experiments. Two-tailed Student’s *t* test was used to determine the statistical significance between different experimental groups, which was set at a value of P < 0.05.

## Results

### The Up-Regulation of H3K27me3 Demethylase JMJD3 Post SCI

**Fig. 1:**
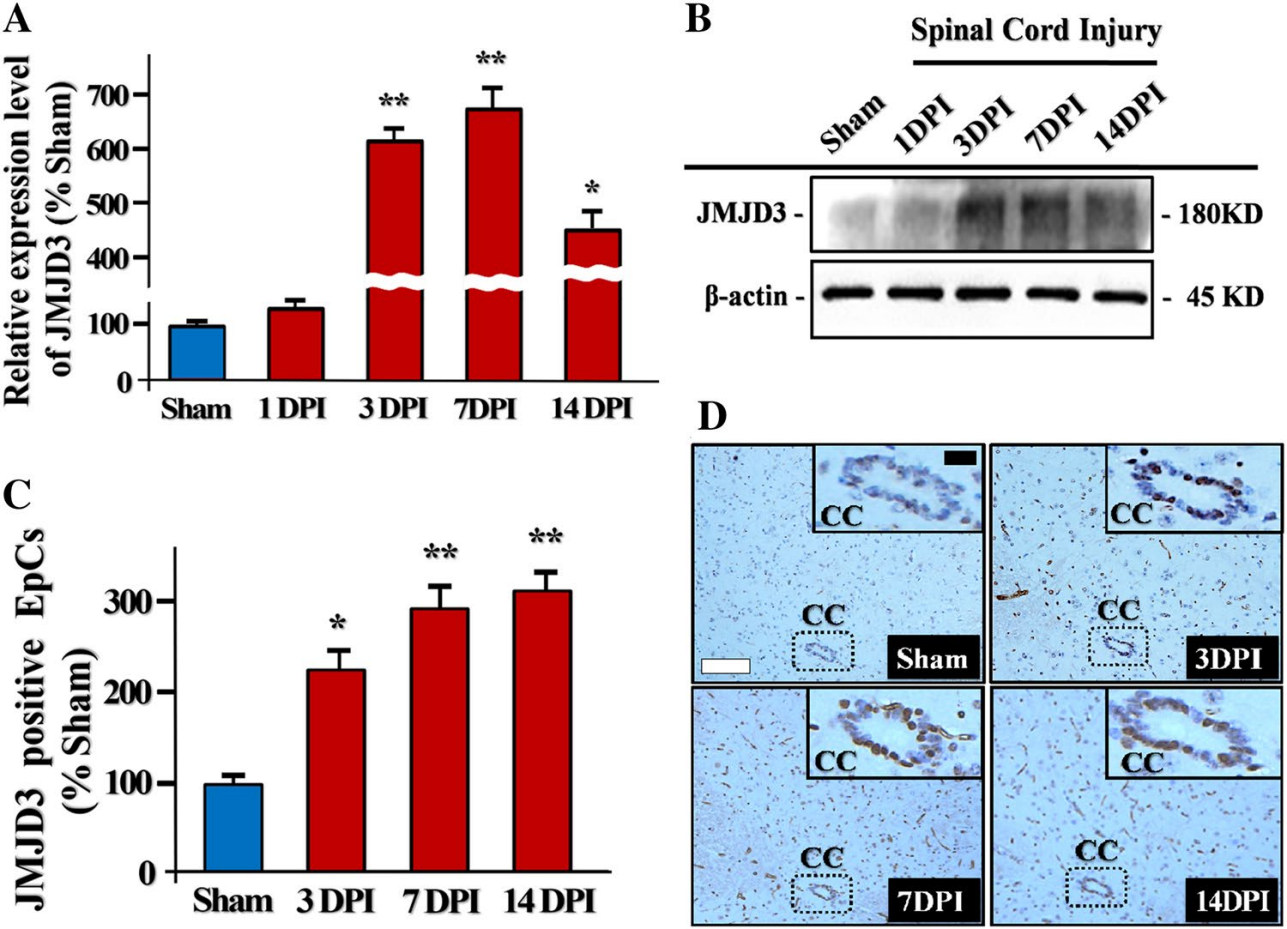
The expression of H3K27me3 demethylase JMJD3 in the SCI epicenter. **a** Quantification of JMJD3 relative expression level at 1 day, 3 days, 7 days and 14 days post SCI, n = 3. **b** Representative immunoblots of JMJD3 expression levels in the sham group and at 1 day, 3 days, 7 days and 14 days post SCI (0.5 cm around contusion epicenter). **c** Representative IHC image of JMJD3 positive EpCs distribution around the central canal zone (CC) at 3 days, 7 days and 14 days post SCI. Of note, the SCI induced JMJD3 positive EpCs increase was discovered around the CC. **d** Quantification of JMJD3 positive EpCs around CC at 3 days, 7 days and 14 days post SCI, n = 6. *: The differences between groups are statistically significant, p < 0.05. **: p < 0.001. Error bars throughout the figure represent the SD (standard deviation) from each independent replicates

To explore the overall fluctuation tendency of H3K27me3 demethylase JMJD3 post SCI (T9 level), we investigated the JMJD3 protein expression level around the injured spinal cord epicenter (0.5 cm around crush site). The SCI secondary injury processes can be divided into three continuous phases chronologically: acute phase (1–2 days), subacute phase (3–7 days) and chronic phase (2 weeks) [11]. Consequently, we set the checkpoints according to the phases. We first found a slight elevation of JMJD3 expression at 1-day post injury (1DPI). Afterwards, JMJD3 expression increased at 3DPI, and then peaking at 7DPI. (Fig. 1a, b). The central canal zone(CC) was regarded as the niche of mitotically quiescent adult NPCs, such as ependymal cells (EpCs), which was proved as the regeneration pool of endogenous cell genesis [18]. We next detected the JMJD3 expression profiles of in spinal cord EpCs around CC post SCI by performing immunohistochemical (IHC) assay. The increase of JMJD3 positive EpCs around CC were observed at 3DPI (Fig. 1c). In addition, the JMJD3 positive EpCs evenly distribute around the CC post SCI (Fig. 1c). Quantitative analysis showed that the number of JMJD3 positive EpCs start to increase significantly since 3DPI (Fig. 1d). Taken together, these results demonstrate that SCI triggers the JMJD3 up-regulation in the spinal cord.

### JMJD3 Inhibition Promotes In Vitro Oligodendrocyte-Lineage Commitment of NPCs

**Fig. 2:**
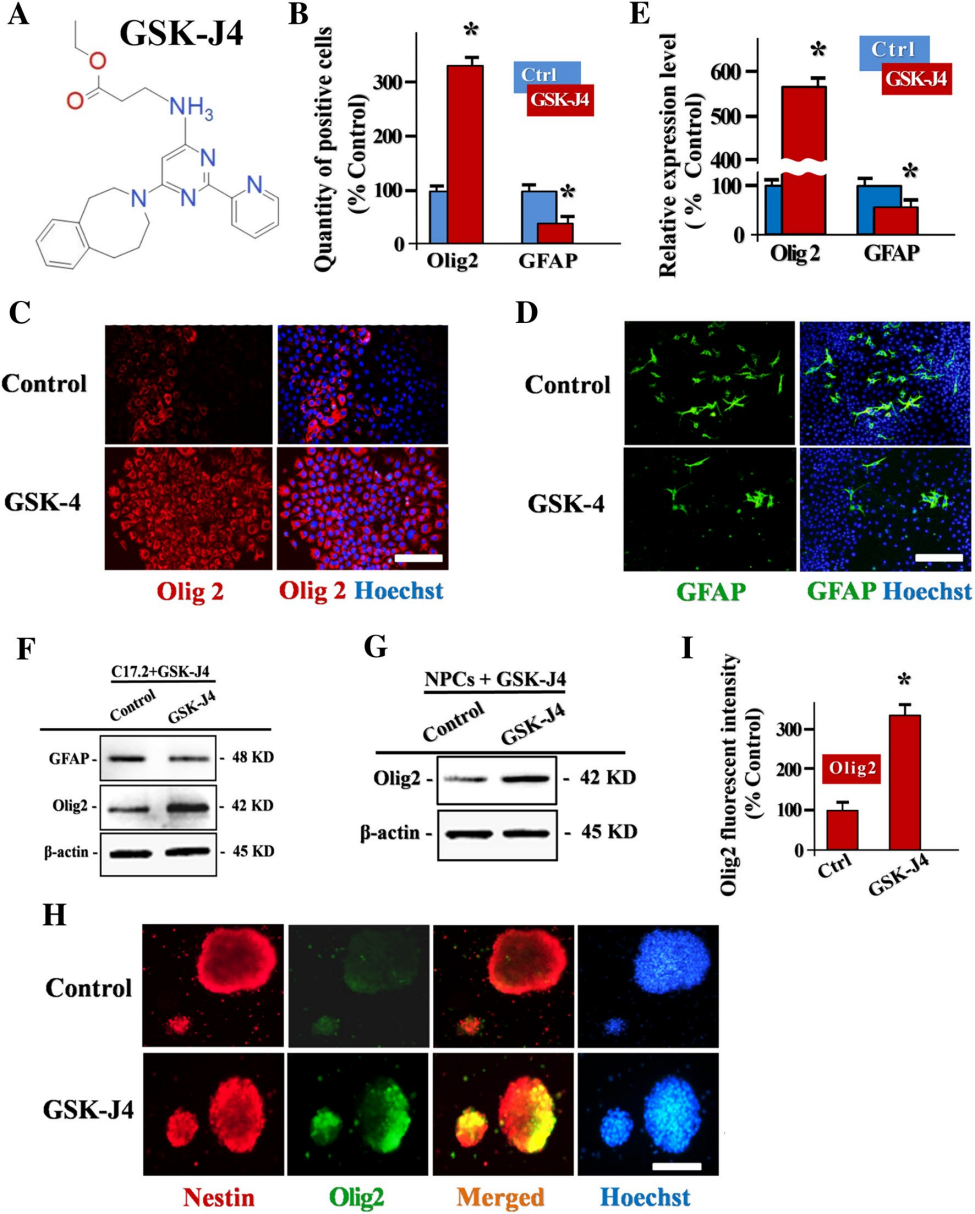
JMJD3 inhibition promotes in vitro oligodendrocyte-lineage commitment of NPCs. **a** The molecular formula of GSK-J4 is C_24_H_27_N_5_O_2_. It is a structure-guided selective chemosynthetic inhibitor of H3K27me3-specific demethylase subfamily. **b** Quantification of Olig2 positive C17.2 cells after 3 days GSK-J4 administrated culture, n = 3. **c** and **d** Representative images of Olig2 (Red) and GFAP (Green) positive C17.2 cells in colonies in GSK-J4 administrated culture system. Scale bar, 50 uM. **e** Quantification of lineage markers Olig2 and GFAP relative expression in C17.2 cells in GSK-J4 administrated culture system, n = 3. **f** Representative immunoblots of lineage markers Olig2 and GFAP in GSK-J4 administrated C17.2 line. **g** Representative immunoblots of lineage markers Olig2 and GFAP in GSK-J4 administrated spinal cord NPCs. **h** Representative immunofluorescent images of Nestin (Red) and Olig2 (Green) expression status in spinal cord NPCs. Note that JMJD3 inhibition results in the Olig2 up-regulation of NPCs. Scale bar, 200 uM. **i** Quantification of relative Olig2 positive florescent intensity of NPC neurospheres post immunofluorescent staining, n = 3. *: The differences between groups are statistically significant, p < 0.001. Error bars throughout the figure represent the SD (standard deviation) from each independent replicates

GSK-J4, the molecular formula is C_24_H_27_N_5_O_2_, is a structure-guided selective Chemosynthetic inhibitor of H3K27me3-specific demethylase [19]. By targeting the JmjC domain, the catalytic center of JMJD3, GSK-J4 regulates the transcription events of H3K27me3 marked genes in an epigenetic manner (Fig. 2a). Consequently, here we conducted JMJD3 inhibitory study in NPCs by employing GSK-J4. We first optimized the GSK-J4 working concentration at 10 uM (6 h), which not only ensure JMJD3 catalytic property, but avoids the compensatory JMJD3 overexpression (Fig. S1, 2). Quantitative analysis demonstrated that GSK-J4 induced JMJD3 inhibition increased the H3K27me3 expression significantly in C17.2 cell line (Fig. S3).

The internal milieu of injured spinal cord is highly hostile for NPCs derived endogenous neurogenesis. Instead, the glial cells are the terminal fate of NPCs differentiation predominately [12, 20]. To elucidate the potential roles of JMJD3 in NPCs lineage specification, the JMJD3 loss of function assay was performed. As a pivotal transcription factor regulate the oligodendrocyte-lineage commitment, Olig2 expression was investigated [3]. In C17.2 cell line, the Olig2 positive cells in the neurospheres largely increased after 2 days GSK-J4 exposure (Fig. 2b, c). Conversely, the GFAP positive cells decreased in the C17.2 neurospheres (Fig. 2b, d). The quantitative analysis demonstrated that the expression level of Olig2 in C17.2 after GSK-J4 treatment was significantly higher than control counterparts (Fig. 2e, f). To further determine whether the JMJD3 activity affects the spinal cord endogenous NPCs specification, we performed the GSK-J4 inhibition culture in spinal cord primary NPCs. Likewise, JMJD3 inhibition also induced the dramatic elevation of Olig2 expression in spinal cord NPCs (Fig. 2g, h). The quantitative analysis of NPCs colony fluorescence intensity demonstrated that Olig2 expression significantly enhanced in GSK-J4 treated group (Fig. 2i). Together, these results revealed that the JMJD3 inhibition in NPCs functionally promotes the oligodendrocyte-lineage commitment.

### JMJD3 Inhibition Promotes the In Vivo Oligodendrocyte-Lineage Commitment Post SCI

**Fig. 3:**
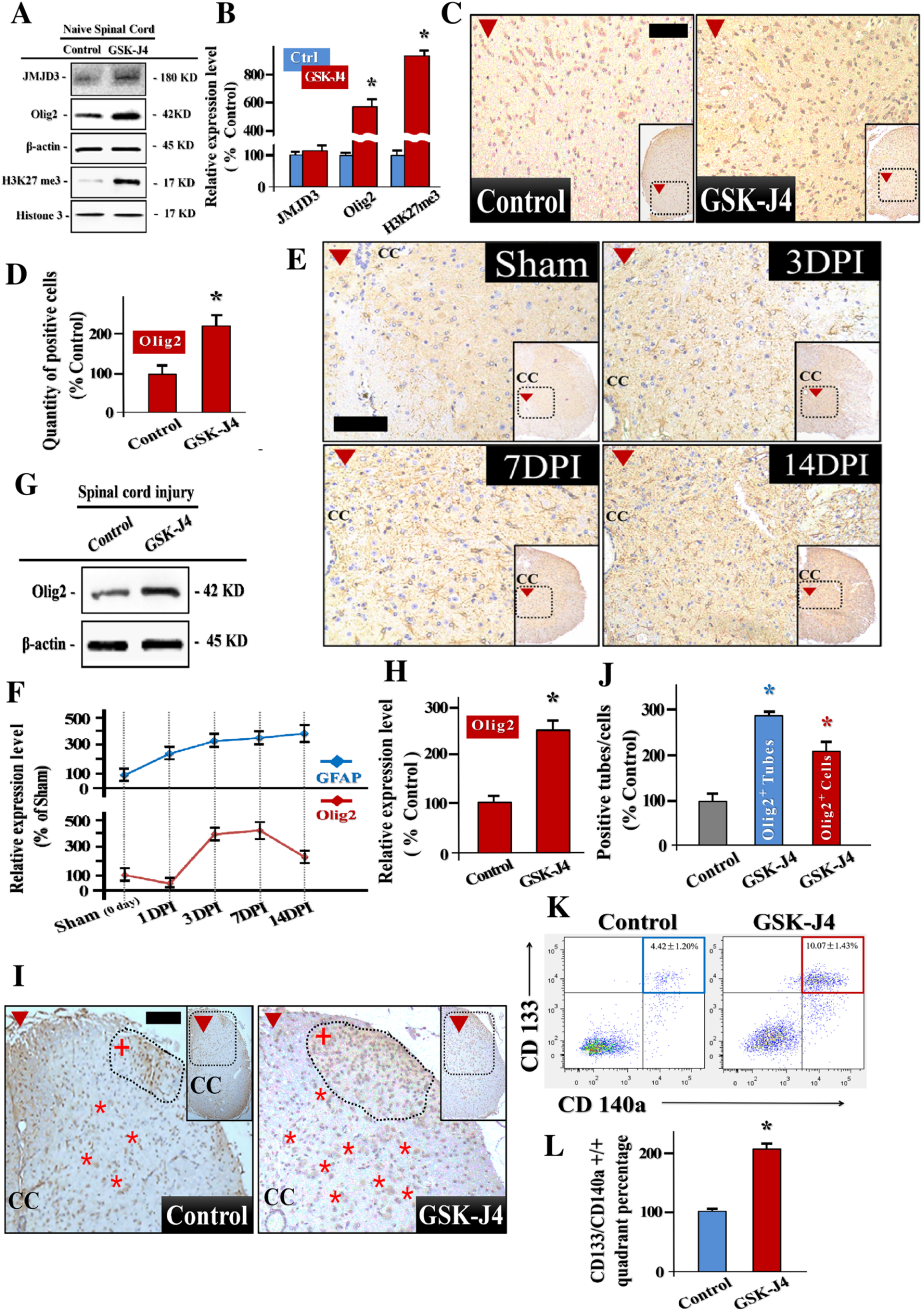
JMJD3 inhibition promotes in vivo oligodendrocyte-lineage commitment post SCI. **a** Representative immunoblots of JMJD3, Olig2 and H3K27me3 in JMJD3 inhibition and control group spinal cords. **b** Quantification of JMJD3, Olig2 and H3K27me3 relative expression levels in inhibition and control group naive spinal cords, n = 6. **c** Representative IHC image of Olig2 positive cells distribution in JMJD3 inhibition and control group spinal cords, central canal zone (CC). **d** Quantification of Olig2 positive cells in naive spinal cord sections in GSK-J4 and control groups, n = 6. **e** Representative immunoblots of Olig2 and GFAP relative expression levels at 1 day, 3 days, 7 days and 14 days post SCI. **f** Quantification of Olig2 and GFAP relative expression levels on these time points post SCI, n = 6. **g** Representative immunoblots of Olig2 in JMJD3 inhibition and control group spinal cords tissue around SCI epicenter. **h** Quantification of Olig2 relative expression in GSK-J4 and control group spinal cords tissue around SCI epicenter, n = 6. **i** Representative IHC image of Olig2 positive cells distribution post SCI in JMJD3 inhibition and control group (Axial sections around spinal cord epicenter). Red*: Olig2 positive cells, Red + : tube-like structures. **j** Quantification of Olig2 positive cells and tube-like structures in GSK-J4 and control group spinal cords tissue around SCI epicenter, n = 6. **k** The oligodendrocyte-lineage associated markers CD133/CD140a in spinal cord lesion post SCI were analyzed by FACS. This is the Representative diagram of FACS assays. **l** Quantification of CD133/CD140a double positive quadrant percentage in GSK-J4 and control group spinal cord lesion post SCI, n = 3. *: The differences between groups are statistically significant, p < 0.001. Error bars throughout the figure represent the SD (standard deviation) from each independent replicates

To extend our in vitro findings to in vivo, we first investigated the influence of JMJD3 inhibition on naive spinal cord. The in vivo JMJD3 inhibition model was established by GSK-J4 injection for 5 days i.p. and 7 days s.c. (Fig. S4). We found that the protein expression of H3K27me3 and Olig2 in spinal cord GSK-J4 injection site increased significantly (Fig. 3a, b). Similar results were obtained in spinal cord section staining, in vivo JMJD3 inhibition results in remarkable Olig2 up-regulation (Fig. 3c, d). We next examined the characteristics of SCI induced Olig2 and GFAP expression fluctuation. We found that expression level of endogenous GFAP increased sharply in the spinal cord lesion post SCI. However, the Olig2 expression dropped significantly at 1 DPI, and followed by a gradually elevation. Finally, the Olig2 expression decreased severely again at 14DPI (Fig. 3e, f).

**Fig. 4:**
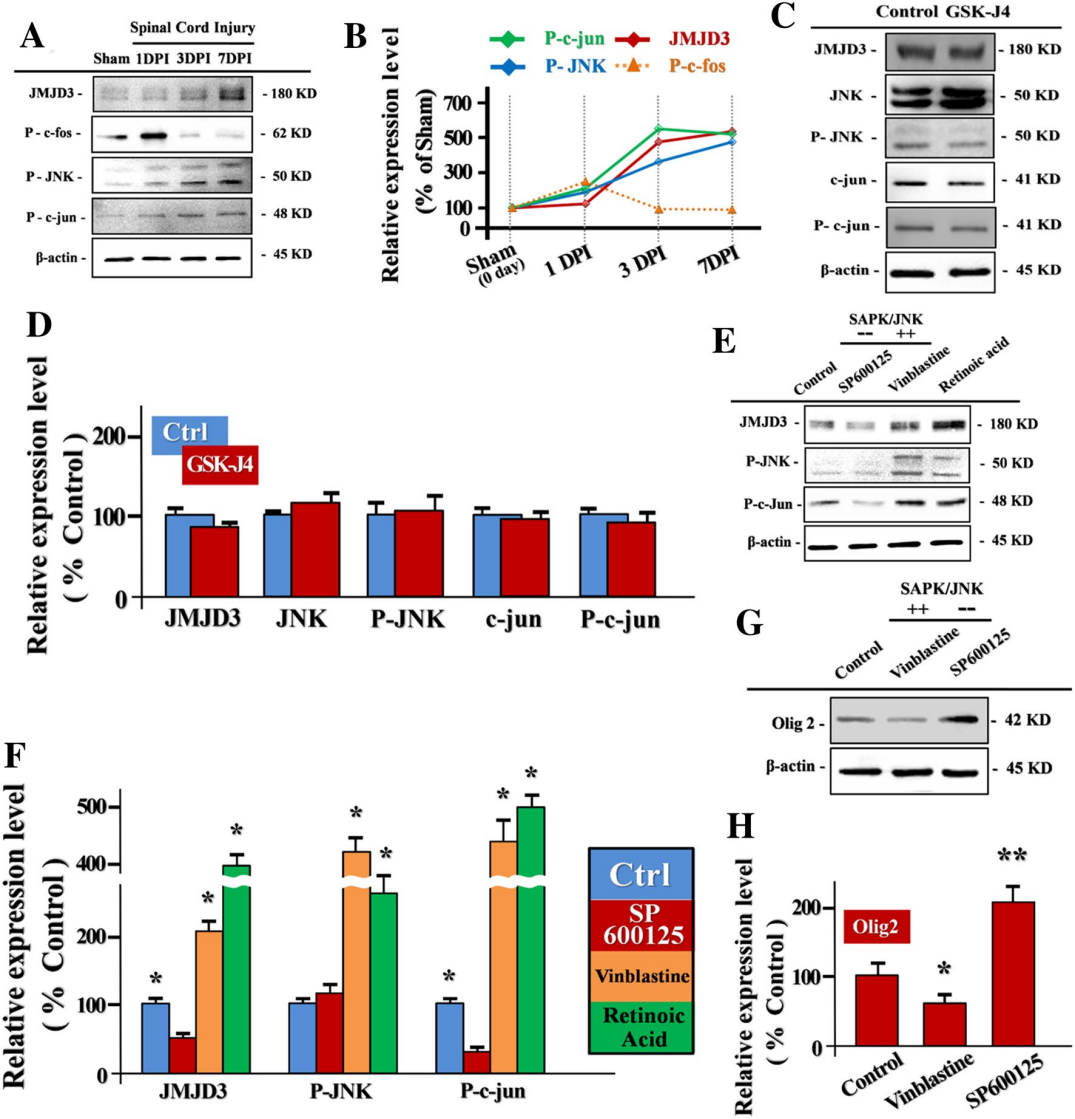
SAPK/JNK signaling pathway targets JMJD3 in NPCs oligodendrocyte-lineage commitment post SCI. **a** Representative immunoblots of JMJD3, P-JNK, P-c-fos and P-c-jun in sham group and at 1 days, 3 days and 7 days post SCI. **b** Quantification of JMJD3, P-JNK, P-c-fos and P-c-jun relative expression levels on these time points post SCI, n = 3. **c** Representative immunoblots of JMJD3 and key factors in SAPK/JNK pathway in control and JMJD3inhibition group. **d** Quantification of JMJD3, P-JNK, P-c-fos and P-c-jun relative expression levels in control and GKS-J4 groups. Note that GSK-J4 intervention does not affects the expression of these key factors in SAPK/JNK pathway, n = 3. **e** Representative immunoblots of JMJD3 and key factors in SAPK/JNK pathway under the DMSO, SP600125, Vinblastine and RA intervention. **f** Quantification of JMJD3, P-JNK, and P-c-jun relative expression levels in groups under the DMSO, SP600125, Vinblastine and RA intervention, n = 3. Note the JMJD3 expression level in NPCs is in parallel with the SAPK/JNK activity. **g** Representative immunoblots of Olig2 expression in control, SP600125 and Vinblastine groups. **h** Quantification of Olig2 relative expression levels in control, SP600125 and Vinblastine groups, n = 3. *: The differences between each group are statistically significant, p < 0.05. Error bars throughout the figure represent the SD (standard deviation) from each independent replicates

To reveal the regulatory effect of JMJD3 on endogenous gliogenesis post SCI, we performed the in vivo JMJM3 loss of function experiment. The JMJD3 inhibition SCI model was established post a five-day GSK-J4 injection (i.p.) for general inhibition JMJD3. Afterward, the injured mice were subjected to GKS-J4 spine wound local injection s.c., and the lesion segment Olig2 expression was assessed 7 days later (Fig. S5). We found that JMJD3 inhibition significantly increase the Olig2 expression level in the spinal cord tissue around epicenter (Fig. 3g, h). To further explore the distribution and the morphology of Olig2 positive cells post SCI, spinal cord sections around epicenter were analyzed. We found that the quantity of Olig2 positive cell in GSK-J4 treatment spinal cords was larger than its control counterparts (Fig. 3i, j). In addition, in the GSK-J4 administrated spinal cords, more Olig2 positive tube-like structures were observed which suggests the remyelination post SCI (Fig. 4j). We next verified the oligodendrocyte-lineage associated markers expression changes in spinal cord lesion post SCI. By utilizing FACS, the CD133/CD140a double positive cells elevation was detected in JMJD3 administrated SCI model (Fig. 3k, l).

Taken together, these findings suggest that the JMJD3 inhibition promotes oligodendrocyte-lineage commitment post SCI.

### SAPK/JNK Signaling Pathway Targets JMJD3 in NPCs Oligodendrocyte-Lineage Commitment Post SCI

Stress-activated protein kinase signaling pathway (SAPK/JNK pathway) plays a critical role in transmembrane signal delivery of cellular stress events, such as mechanical traumatic stress and UV radiation damage [21, 22]. Emerging evidences suggests that SAPK/JNK pathway involved in the regulation of stem cell metabolism, differentiation and survival in CNS [23–26]. However, the function of SAPK/JNK signaling in SCI associated epigenetic regulation yet to be unfolded.

Therefore, we first detected that the expression of phosphorylated JNK (P-JNK, Thr183/Try185) elevated gradually post SCI, which indicates the SAPK/JNK pathway was activated. Importantly, the similar expression trends of P-JNK and JMJD3 in 1-week post SCI suggest that the SAPK/JNK signaling activation closely related to the chronological up-regulation of endogenous JMJD3 post SCI (Fig. 4a, b).

To verify the latent downstream targets of SAPK/JNK pathway activation post SCI, we tested the phosphorylation level of AP-1 subunits, c-jun and c-fos. We found that the phosphorylated c-jun (P-c-jun, Ser63) level increased gradually post SCI which was paralleled to the P-JNK expression (Fig. 4a, b). By contrast, the P-c-fos expression presented an early phase elevation post SCI (Fig. 4a, b). The result showed that the c-jun acts as the downstream molecular of SAPK/JNK pathway in SCI signal transduction.

To further make sure the correlations between JMJD3 and SAPK/JNK pathway, we analyzed the SAPK/JNK pathway key factors expression profiles in C17.2 NPCs line. We found that the inhibition of JMJD3 caused no significant effect on either the activity or the expression of those key factors in SAPK/JNK pathway (Fig. 4c, d). The result suggested that JMJD3 is a potential downstream target of SAPK/JNK pathway.

We next investigated if SAPK/JNK pathway regulates the JMJD3 expression in NPCs by manipulating the SAPK/JNK signaling activity. In this section, reagents such as SP600125, Vinblastine and Retinoic acid were employed to detect the SCI associated signaling pathway. SP600125 is a reversible JNK inhibitor which inhibits the phosphorylation of the JNK downstream target c-Jun. Vinblastine is an agonist of JNK which maintaining the JNK signaling pathway activation by strongly increasing both the expression and phosphorylation of c-Jun. Retinoic acid was demonstrated can induce the NPCs differentiation and the H3K27me3 down-regulation. We found that the specific inhibitor SP600125 (20 uM, 3 h) induced JNK inactivation in cultured C17.2 NPCs line led to markedly decreased level of JMJD3. By contrast, the activation of JNK induced by Vinblastine (10 uM, 3 h) promotes the expression of JMJD3 in NPCs which was similar to Retinoic acid induced JMJD3 up-regulation [27] (Fig. 4e, f). Interestingly, the Olig2 expression in NPCs was significantly enhanced in SP600125 group compared with that in Vinblastine treatment counterparts (Fig. 4g, h).

Collectively, the results demonstrated that JMJD3 downstream of SAPK/JNK signaling pathway in regulating the NPCs oligodendrocyte-lineage commitment post SCI.

## Discussion

SCI induced neurological disability is a nightmare to both patients and doctors. The injury induced OLs loss and demyelination occurred immediately post SCI, which disrupt the neural circuits destructively [28]. (Fig. S6) Therefore, effective interventions to promote the spontaneous OLs replacement will be bound to cross the intrinsic barrier to SCI recovery [10, 29]. During the gliogenesis process post injury, promoting the oligodendrocyte-lineage commitment is the precondition of NPCs derived endogenous oligodendrocytes regeneration [12]. Even so, powerful strategy is yet to be discovered. Epigenetic modifications are emerging as critical molecular switch of gene expression, which were expected to manipulate the regeneration potential of adult mammalian CNS [30–32]. However, the molecular mechanisms underlying the interplay between epigenetic network and OLs post SCI remains elusive.

JMJD3 mediated H3K27me3 demethylation is a critical repressive epigenetic event, which exerts functions in multiple physiological and pathological processes [33, 34]. Although previous study have shown that JMJD3 deeply involved in the neurogenesis and neurodegeneration of CNS, as cellular responses to SCI, the underlying epigenetic motivations of JMJD3 fluctuation still to be further investigated [34, 35]. From the pathological gliogenesis perspective, the overwhelming majority of newly generated cells in response to SCI are reactive astrocytes [36].

From the pathological gliogenesis perspective, the overwhelming majority of newly generated cells in response to SCI are reactive astrocytes [36]. Glial fibrillary acidic protein (GFAP) is an intermediate filament enriched in astrocytes [37]. Here, we found the up-expression of GFAP in spinal cord lesion, which indicates the proliferation of the reactive astrocytes induced by SCI (Fig. 3e, f). These in vivo changes of GFAP post SCI are in accordance with the role astrocytes played during the nature course of SCI pathological repairing. At the beginning of SCI, reactive astrocytes played a positive role in SCI epicenter by limiting the inflammation dissemination. However, later, the secondary SCI injury induced inflammation cascades turn the lesion into a hostile environment which hinder the axon to grow across. Consequently, either the astrocyte-lineage commitment or the proliferation of reactive astrocyte of NPCs post SCI impede the neurological recovery eventually.

In this study, the Olig2 up-regulation was detected post JMJD3 inhibition in SCI lesion (Fig. 3g, e). Even if the accurate mechanism of NPCs fate transition is unknown, this result suggests that the JMJD3 inhibition induced H3K27me3 enrichment state during endogenous gliogenesis is likely to modulates appropriate epigenetic backgrounds for OLs lineage specification post SCI. Additionally, it is presumable that there is a strong relationship between SCI triggered JMJD3 accumulated state and astrocyte-lineage commitment during the nature course of SCI pathological repair. Interestingly, recent studies discovered several demethylase property independent functions of JMJD3 in various cell types [38, 39]. Here we found that JMJD3 inhibition promotes the elevation of H3K27me3 in NPCs. Under such condition, the Olig2 expression increased in parallel with the H3K27me3 up-regulation (Fig. S1–2). Consequently, our finding suggests that the JMJD3 regulates the Olig2 expression in NPCs in an epigenetic mechanism dependent manner.

Here, we also provide strong and novel evidence that JMJD3 downstream of SAPK/JNK pathway in SCI traumatic signaling transduction. In particular, SAPK/JNK inactivation promotes the Olig2 up-regulation in NPCs (Fig. 4g). SAPK/JNK signaling pathway is essential for evoking the cellular response to stress events [40, 41]. However, as protein kinases pathway, neither JNK nor its down target AP-1 is capable to rewrite epigenetic marks in nucleus directly, such as H3K27me3. Base on the results, we speculate that JMJD3 functions as decoder which translates the SCI associated stress signaling into nucleus readable epigenetic codes.

Previous Studies illustrated that more than 90% OLs around the epicenter were lost within the first week post SCI [6]. Accordingly, promoting OLs regeneration earlier post SCI is of great significance to rescue the demyelination and subsequent axon degeneration. Here, we found that JMJD3 expression start to elevated post injured, and peaked at 7 DPI. Additionally, JMJD3 inhibition promotes the Olig2 positive cells accumulation in SCI lesion. Importantly, in GSK-J4 administrated spinal cords, morphological characteristics of myelinating oligodendrocytes such as ramified processes and tube-like structures can be recognized in Olig2 positive cells (Fig. 3i–k). These findings suggest that the JMJD3 inhibition is required for early oligodendrocyte-lineage commitment awakening post SCI.

H3K27me3 is a well-documented repressive epigenetic hallmark which was dynamically catalyzed by and methylase and demethylase [16]. Studies suggested that surplus H3K27me3 expression is required for pluripotency state maintenance of stem cells [42]. However, these results seem contradictory to what we showed here. Although, the precise mechanism still unclear, one likely reason for the paradoxical results is the bivalent mark phenomenon of key lineage genes. Structurally, the bivalent domains are shaped by two elements, the repressive unit H3K27me3, and the permissive unit H3K4me3. These units enable the conjugated and precise spatial manipulation of lineage decision transcription factors in stem cells [43, 44]. Recent study discovered an alternate enrichment of H3K27me3 and H3K4me3 at the same RAGs promoter regions that during CNS development [45]. Here, we detected that JMJD3 inhibition induced H3K27me3 up-regulation promotes Olig2 expression in SCI lesion. From this point, the Olig2 up-regulation results from the Histone3 bivalent domains mediated epigenetic background modification. Thus, it suggests that certain inhibitory genes against oligodendrocyte-lineage selection might be blocked by JMJD3 inhibition mediated H3K27me3 gene silencing. Nevertheless, further evidence will be imperative to elucidate the epigenetic mechanism of H3K27me3 mediated Olig2 expression.

Taken together, we demonstrated that JMJD3 inhibition mediated SAPK/JNK signaling inactivation is functionally necessary for endogenous oligodendrocyte-lineage commitment post SCI. Importantly, these findings would be a novel epigenetic approach to boost the mature mammalian endogenous recovery post SCI.

## Supplementary information

Below is the link to the electronic supplementary material.

Supplementary material 1 (TIFF 6192 kb) (S1) Representative immunoblots of GSK-J4 working concentration optimization in C17.2 line. Note that the 10 uM/6hours GSK-J4 condition is ideal for JMJD3 inhibition in NPCs. (S2) Representative immunofluorescent image of JMJD3 and H3K27me3 in spinal cord NPCs in control and GSK-J4 group, Scale bar, 200 uM. (S3) Quantification of JMJD3 and H3K27me3 relative expression in NPCs post JMJD3 inhibition. Note that GSK-J4 induced JMJD3 inhibition increased the H3K27me3 expression significantly. *: The differences between each group are statistically significant, p<0.001. Error bars throughout the figure represent the SD (standard deviation) from each independent replicates. (S4) Schematics of the protocol for in vivo JMJD3 inhibition mice model. (S5) Schematics of the protocol for in vivo JMJD3 inhibition SCI model. (S6) Representative IHC image of Olig2 positive cells in the spinal cord post SCI in WT mice (Sagittal sections around spinal cord epicenter).

## Abbreviations

SCI: Spinal cord injury
OLs: Oligodendrocytes
CNS: Central nervous system
H3K27me3: Tri-methylation of lysine27 on histone3
JMJD3: Jmjc-domain containing histone demethylase
NPCs: Neural stem/progenitor cells
CC: Central canal
IHC: Immunohistochemical
EpCs: Ependymal cells
SAPK/JNK pathway: Stress-activated protein kinase signaling pathway
RA: Retinoic acid
