# Supplementary figures and images for: Unlocking the Recovery Potential: JMJD3 Inhibition-Mediated SAPK/JNK Signaling Inactivation Supports Endogenous Oligodendrocyte-Lineage Commitment Post Mammalian Spinal Cord Injury
Source: Neurochem Res. 2021 Jan 11;46(4):792–803. doi: 10.1007/s11064-020-03210-z (PMC7946673; doi:10.1007/s11064-020-03210-z)

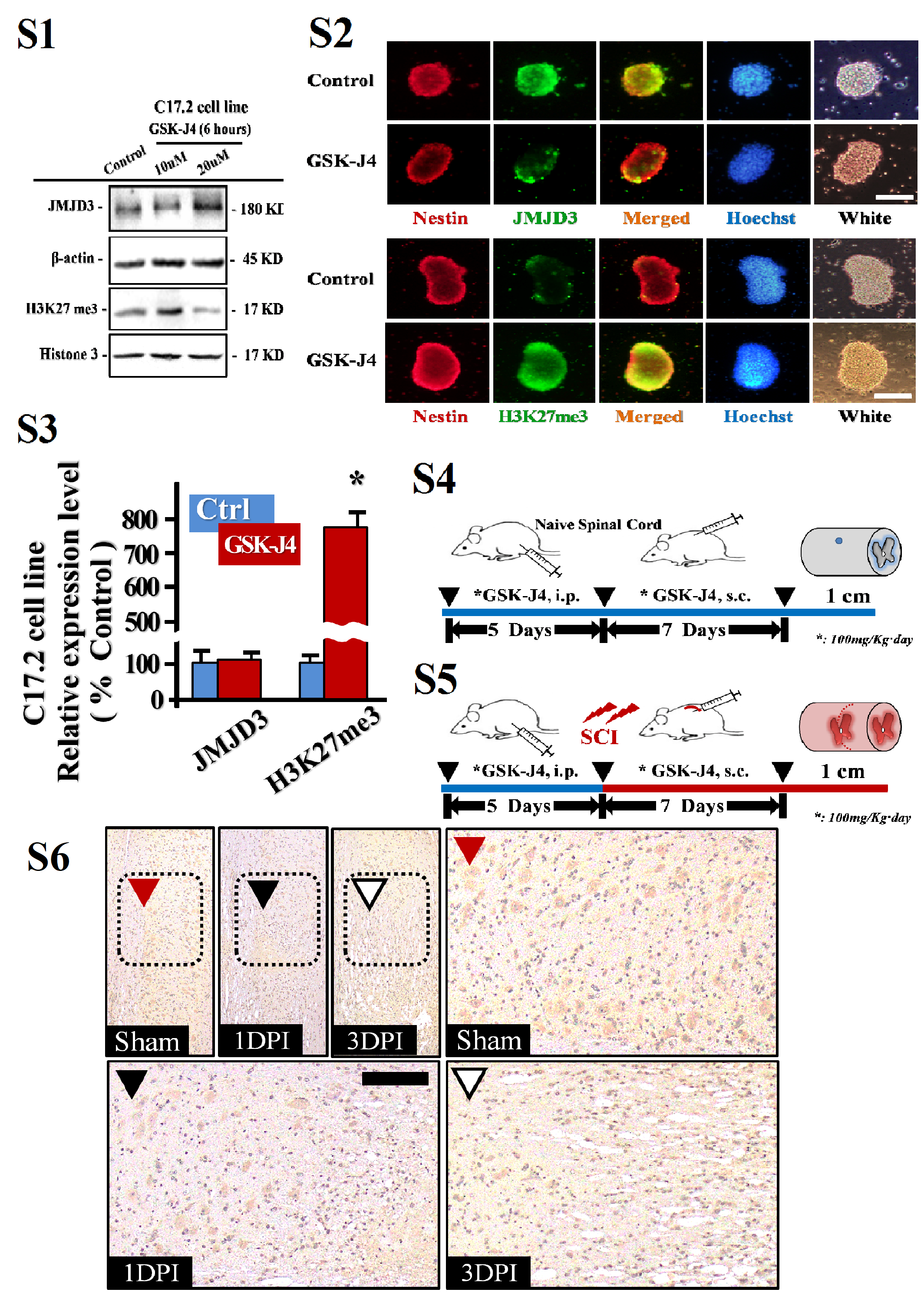

Supplement: Supplementary file 1 — Supplementary material 1 (TIFF 6192 kb) (S1) Representative immunoblots of GSK-J4 working concentration optimization in C17.2 line. Note that the 10 uM/6hours GSK-J4 condition is ideal for JMJD3 inhibition in NPCs. (S2) Representative immunofluorescent image of JMJD3 and H3K27me3 in spinal cord NPCs in control and GSK-J4 group, Scale bar, 200 uM. (S3) Quantification of JMJD3 and H3K27me3 relative expression in NPCs post JMJD3 inhibition. Note that GSK-J4 induced JMJD3 inhibition increased the H3K27me3 expression significantly. *: The differences between each group are statistically significant, p<0.001. Error bars throughout the figure represent the SD (standard deviation) from each independent replicates. (S4) Schematics of the protocol for in vivo JMJD3 inhibition mice model. (S5) Schematics of the protocol for in vivo JMJD3 inhibition SCI model. (S6) Representative IHC image of Olig2 positive cells in the spinal cord post SCI in WT mice (Sagittal sections around spinal cord epicenter). [file 11064_2020_3210_MOESM1_ESM.tif]
